# Time-dependent diffusion in one-dimensional disordered media decorated by permeable membranes: Theoretical findings backed by simulations and a new disorder class

**DOI:** 10.1063/5.0272370

**Published:** 2025-06-27

**Authors:** Magnus Herberthson, Peter J. Basser, Evren Özarslan

**Affiliations:** 1 Department of Mathematics, Linköping University, Linköping, Sweden; 2 Spin Nord AB, Linköping, Sweden; 3 Section on Quantitative Imaging and Tissue Sciences, Eunice Kennedy Shriver National Institute of Child Health and Human Development, NIH, Bethesda, Maryland 20892, USA; 4 Department of Biomedical Engineering, Linköping University, Linköping, Sweden

## Abstract

As the diffusion of fluids is hindered by semipermeable membranes, the long-time behavior of the diffusion coefficient is influenced by the arrangement of the membranes. We develop methods that predict this long-time instantaneous diffusivity from bulk diffusivity, the membranes' locations, and their permeabilities. We studied this problem theoretically and expressed the instantaneous diffusivity analytically as an infinite sum. An independent numerical scheme was employed. Several types of disorder in the membranes' positions were considered including a new disorder family that generalizes hyperuniform and short-range disorders. Our theoretical and numerical findings are in excellent agreement. Our methods provide an alternative means for studying time-dependent diffusion processes.

## I. INTRODUCTION

The behavior of diffusing particles is deeply influenced by the structural organization of the medium, with variations in order or disorder leaving measurable imprints on diffusion dynamics.^1,2^ The diffusion coefficient of fluids is a key parameter that provides insight into the structural and dynamical properties of the surrounding medium. Its value and time-dependent behavior are strongly influenced by the medium's microstructural characteristics. Specifically, how the diffusion coefficient evolves over time encodes valuable information about the geometric constraints that hinder the diffusive motion. The feasibility of observing time-dependent diffusion via magnetic resonance (MR) methods has attracted a great deal of interest in studying time-dependent diffusion in porous media, in particular, within tissues.^3^

Previous studies in the field of porous media have established that the diffusion coefficient exhibits distinct behaviors at different timescales. In the short-time regime, its evolution is primarily governed by the surface-to-volume ratio of the confining medium.^4–7^ This dependence arises because, at very short times, diffusing molecules predominantly experience interactions with nearby barriers rather than experience organizational features of the structure at longer length scales.

In contrast, in the long-time limit, the diffusion coefficient is determined by the tortuosity and characteristic length scales of the microstructural network.^8^ At sufficiently long observation times, diffusing molecules effectively sample large domains, making the macroscopic diffusion properties sensitive to the connectivity and complexity of the medium.

This long-time behavior is particularly relevant to understanding the organization and structure of biological tissues, where cells and extra-cellular space are separated by semipermeable membranes, which play critical roles in shaping the observed diffusion dynamics.^9^ Unlike purely impermeable barriers, biological membranes allow for exchange, i.e., a transition between compartments. This exchange process, coupled with the characteristic size of cells, the bulk water diffusivity, and the timescales accessible via measurements, determines both the long-time asymptote of the diffusion coefficient and the manner in which this asymptote is approached.^6,10^

Of particular interest is the functional form of the approach to the long-time limit, as it can provide crucial insights into the type and degree of disorder present in the system. Different structural arrangements, such as ordered lattices,^8,11,12^ fractal-like geometries,^13,14^ or packed random barriers,^15^ can give rise to distinct scaling laws governing the decay of diffusivity as a function of diffusion time. Consequently, detailed analysis of the long-time behavior of the diffusivity may serve as a powerful tool for probing microstructural heterogeneity and distinguishing between or among different tissue architectures in both healthy and pathological conditions.

Here, we focus on a one-dimensional scenario for simplicity. Earlier studies on one-dimensional disordered media have provided a range of analytical tools and results for understanding transport and reaction-diffusion dynamics.^16^ The low-frequency behavior of disordered diffusion models with divergent hopping distributions has been characterized in terms of the density of states, diffusion constant, and localization length, confirming key scaling assumptions.^17^ Reaction-diffusion models with quenched disorder featuring disconnected domains of reaction have been extensively investigated, and exact solutions for the long-time behavior of density and correlation functions were obtained.^18^ For diffusion with random local bias, real-space renormalization group methods have yielded detailed asymptotic descriptions of particle densities, persistence, and critical exponents associated with dynamical phase transitions.^19^ Complementary approaches such as the effective medium approximation have revealed multiple temporal and thermal regimes of the diffusion coefficient in energy landscapes featuring chains of wells with random depths or barriers with random heights.^20^ On a more general level, fractional kinetic equations, derived from random walk and generalized master equations, have emerged as a versatile framework to capture anomalous transport and non-exponential relaxation in complex disordered systems.^21^

**FIG. 1.**
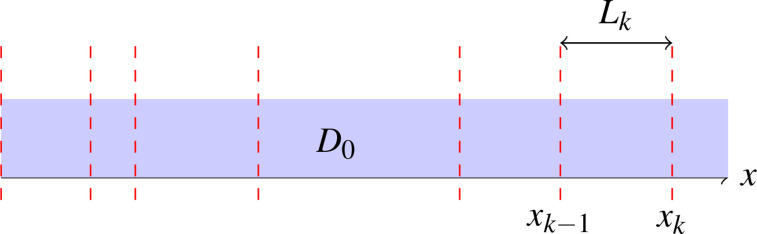
Diffusion occurs within a one-dimensional domain separated by thin membranes with permeability *P*. The bulk diffusivity of the fluid is $D_{0}$. The *k*th interval, having length $L_{k}$, is located between the points $x_{k-1}$ and $x_{k}$.

We shall consider a one-dimensional medium characterized by a random distribution of infinitesimally thin membranes having permeability *P* as illustrated in Fig. 1. The average spacing between the neighboring membranes is denoted by *L*. As the diffusion time increases, the diffusivity of the fluid starts from the bulk diffusivity $D_{0}$ and gradually reaches its long-time value, $D_{\infty }$, which is given by Ref. 22,

$$D_{\infty }=D_{0}\frac{LP}{D_{0}+LP}.$$ (1)

In this paper, we study the long-time regime. We propose to employ a convenient and computationally efficient numerical procedure to predict the distribution of particles for any diffusion time. For the long-time regime, we describe how the above infinite-time diffusivity is reached. In this regime, the distribution of the particles starting from a given point is nearly Gaussian^23^ with the time-dependent variance $\sigma^{2}(t)=2\Delta (t)$. By Ansatz, $\Delta (t)=D_{\infty }t+\beta (t)$, and the derivative of Δ(t) is the instantaneous diffusivity, $D_{\text{inst}}(t)$. Hence, the deviation from $D_{\infty }$ is $\beta^{\prime }(t)$, i.e.,

$$D_{\text{inst}}(t)=\Delta^{\prime }(t)=D_{\infty }+\beta^{\prime }(t).$$ (2)

The main theoretical finding of this article is the following functional equation for the unknown function β(t):

$$\frac{\beta^{\prime }(t)}{D_{\infty }}=\frac{D_{0}}{D_{0}+LP}\left(1-\sum_{k=-\infty }^{\infty }\frac{x_{k}^{2}L}{4\sqrt{\pi }{\left(\Delta (t)\right)}^{3/2}}e^{-\frac{x_{k}^{2}}{4\Delta (t)}}\right),$$ (3)

which allows us to study the long-time behavior of the instantaneous diffusivity for any^24^ set of membrane positions, $x_{k}$ (with k=…,−2,−1,0,1,2,…). The formulation of Eq. (3) also allows us to study disordered media wherein these positions are randomly distributed; we also consider scenarios involving hyperuniform, short-range, and strong disorders.^15^ Furthermore, we introduce a new family of disorders wherein the hyperuniform and short-range disorders are special cases.

In Sec. II, we detail our theoretical approach and derive Eq. (3). We describe the new family of disorders in Sec. III. In Sec. IV, we present various results and compare them with findings obtained via a finite-difference framework described in Appendix B.

## II. THEORY

**FIG. 2.**
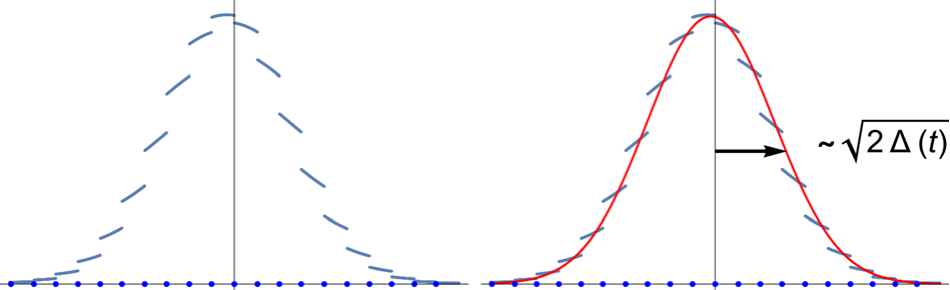
A conceptual sketch of the employed model. For a particle initially at the origin, the distribution $u_{0}(x,t)$ of the position for the particle at a later time *t* would appear as shown on the left. The discontinuities correspond to the presence of membranes. For long times, $u_{0}(x,t)$ has an approximately Gaussian distribution as illustrated via the red curve on the right. The halved variance of this Gaussian is denoted by Δ(t) whose time derivative yields the instantaneous diffusivity, $D_{\text{inst}}(t)$.

We study the long-time dynamics of the quantity $u_{0}(x,t)$, which is the probability that a particle initially at the origin traverses to position *x* over a time-interval *t*. The ansatz is that this quantity is well-represented by a Gaussian distribution at long times^23^ as illustrated in Fig. 2. To simplify the notation in this section, we let $u_{0}^{\prime }(x,t)$ denote its derivative with respect to *x*. [That is, for a function *f* of two variables, $f_{x}^{\prime }(x,t)=\partial_{x}f(x,t)$ is written $f^{\prime }(x,t)$ and similarly for $f_{xx}^{\prime \prime }(x,t)=\partial_{x}^{2}f(x,t)$, which we write as $f^{\prime \prime }(x,t)$. For a function of one variable, no deviant notation is employed.] The condition at a thin membrane can then be written as

$$u_{0}(x_{k}^{+},t)-u_{0}(x_{k}^{-},t)=\frac{D_{0}}{P}u_{0}^{\prime }(x_{k},t).$$ (4)

Although $u_{0}$ is discontinuous, and hence not differentiable at $x_{k}$, its spatial derivative has a limit when $x\to x_{k}$ as $\underset{x\to x_{k}^{-}}{\text{lim}}u_{0}^{\prime }(x,t)=\underset{x\to x_{k}^{+}}{\text{lim}}u_{0}^{\prime }(x,t)$. For convenience, we denote this limit as $u_{0}^{\prime }(x_{k},t)$. Since $u_{0}$ is a probability distribution, it integrates to 1, i.e.,

$$\begin{array}{c} 1=\int_{-\infty }^{\infty }u_{0}(x,t)dx=\sum_{k=-\infty }^{\infty }\int_{x_{k}}^{x_{k+1}}u_{0}(x,t)dx\\ =\sum_{k=-\infty }^{\infty }{\left[xu_{0}(x,t)\right]}_{x_{k}}^{x_{k+1}}-\sum_{k=-\infty }^{\infty }{\left[\frac{x^{2}}{2}u_{0}^{\prime }(x,t)\right]}_{x_{k}}^{x_{k+1}}\\ \;+\sum_{k=-\infty }^{\infty }\int_{x_{k}}^{x_{k+1}}\frac{x^{2}}{2}u_{0}^{\prime \prime }(x,t)dx, \end{array}$$ (5)

where we employed integration-by-parts twice. We split the first two summations into two terms and employ the change k+1→k, which allows us to see that the second summation vanishes, while the first becomes $-D_{0}P^{-1}\sum_{k=-\infty }^{\infty }x_{k}u_{0}^{\prime }(x_{k},t)$ by virtue of the boundary condition, Eq. (4). Finally, we invoke the diffusion equation (in the interior of each interval) in the last summation and find

$$-\frac{D_{0}}{P}\sum_{k=-\infty }^{\infty }x_{k}u_{0}^{\prime }(x_{k},t)+\frac{1}{2D_{0}}\partial_{t}\int_{-\infty }^{\infty }x^{2}u_{0}(x,t)dx=1.$$ (6)

We shall denote the Gaussian that approximates the distribution, as alluded to above, by u(x,t), i.e.,

$$u(x,t)=\frac{1}{\sqrt{4\pi \Delta (t)}}e^{-\frac{x^{2}}{4\Delta (t)}},$$ (7)

which integrates to unity, as well. Moreover, it satisfies the equation

$$\frac{\partial u(x,t)}{\partial t}=\Delta^{\prime }(t)\;u^{\prime \prime }(x,t).$$ (8)

In order for u(x,t) to be a good approximation, its variance must match that of $u_{0}(x,t)$, giving rise to the condition

$$\int_{-\infty }^{\infty }x^{2}u_{0}(x,t)dx=2\Delta (t),$$ (9)

which, along with Eq. (2), can be substituted into Eq. (6) to yield

$$-\frac{D_{0}}{P}\sum_{k=-\infty }^{\infty }x_{k}u_{0}^{\prime }(x_{k},t)+\frac{D_{\infty }+\beta^{\prime }(t)}{D_{0}}=1.$$ (10)

Furthermore, in order for the two distributions to evolve in a consistent manner, the probability of finding a particle within each interval should decay consistently. That is, for any *k*,

$$\frac{\partial }{\partial t}\int_{x_{k}}^{x_{k+1}}u_{0}(x,t)dx=\frac{\partial }{\partial t}\int_{x_{k}}^{x_{k+1}}u(x,t)dx.$$ (11)

Swapping the order of partial differentiation and integration and using the diffusion equation and Eq. (8), we obtain

$$D_{0}\int_{x_{k}}^{x_{k+1}}u_{0}^{\prime \prime }(x,t)dx=\int_{x_{k}}^{x_{k+1}}\Delta^{\prime }(t)\;u^{\prime \prime }(x,t)dx.$$ (12)

Upon explicit integration, we obtain

$$D_{0}\left(u_{0}^{\prime }(x_{k+1},t)-u_{0}^{\prime }(x_{k},t)\right)=\Delta^{\prime }(t)\left(u^{\prime }(x_{k+1},t)-u^{\prime }(x_{k},t)\right).$$ (13)

By adding to the aforementioned equation to the corresponding expressions for the M−1 neighboring intervals to the right, one obtains

$$D_{0}\left(u_{0}^{\prime }(x_{k+M},t)-u_{0}^{\prime }(x_{k},t)\right)=\Delta^{\prime }(t)\left(u^{\prime }(x_{k+M},t)-u^{\prime }(x_{k},t)\right).$$ (14)

As M→∞ (for fixed *t*), both $u_{0}^{\prime }(x_{k+M},t)$ and $u^{\prime }(x_{k+M},t)$ tend to 0, yielding the key expression describing our model,

$$\begin{array}{c} D_{0}u_{0}^{\prime }(x_{k},t)=\Delta^{\prime }(t)u^{\prime }(x_{k},t)=(D_{\infty }+\beta^{\prime }(t))u^{\prime }(x_{k},t). \end{array}$$ (15)

Employing Eq. (15) in Eq. (10), we arrive at the relationship

$$\frac{D_{\infty }+\beta^{\prime }(t)}{D_{0}}-\frac{D_{\infty }+\beta^{\prime }(t)}{LP}\sum_{k=-\infty }^{\infty }Lx_{k}u^{\prime }(x_{k},t)=1.$$ (16)

In Appendix A, we prove that the following statement holds:

$$\sum_{k=-\infty }^{\infty }Lx_{k}u^{\prime }(x_{k},t)=-1+o(1)\;\text{as}\;t\to \infty .$$ (17)

Note that as t→∞, $\beta^{\prime }(t)\to 0$. Thus, to leading order, Eq. (16) becomes

$$1=\frac{D_{\infty }}{D_{0}}+\frac{D_{\infty }}{LP},$$ (18)

which is the well-known expression by Crick^22^ [Eq. (1)].

Since $\beta^{\prime }(t)o(1)=o(\beta^{\prime }(t))$ as t→∞, we can approximate

$$\beta^{\prime }(t)\sum_{k=-\infty }^{\infty }Lx_{k}u^{\prime }(x_{k},t)=-\beta^{\prime }(t)+o(\beta^{\prime }(t)),$$ (19)

and to leading order, Eq. (16) becomes

$$\frac{D_{\infty }+\beta^{\prime }(t)}{D_{0}}+\frac{\beta^{\prime }(t)}{LP}-\frac{D_{\infty }}{LP}\sum_{k=-\infty }^{\infty }Lx_{k}u^{\prime }(x_{k},t)=1.$$ (20)

We now rewrite (20) as

$$\begin{array}{c} \frac{\beta^{\prime }(t)}{D_{0}}+\frac{\beta^{\prime }(t)}{LP}=1-\frac{D_{\infty }}{D_{0}}+\frac{D_{\infty }}{LP}\sum_{k=-\infty }^{\infty }Lx_{k}u^{\prime }(x_{k},t)\\ =\frac{D_{\infty }}{L\;P}+\frac{D_{\infty }}{LP}\sum_{k=-\infty }^{\infty }Lx_{k}u^{\prime }(x_{k},t), \end{array}$$ (21)

where we employed Eq. (1) in the last step.

This means that after differentiation of u(x,t), Eq. (20) becomes

β′(t)D∞=D0D0+LP(1−∑k=−∞∞xk2L4π(Δ(t))3/2e−xk24Δ(t)). (22)

*Remark.* In the expression earlier, we must evaluate $\Delta (t)=D_{\infty }t+\beta (t)$, where β(t) is yet unknown. This is not problematic, however, since $\beta^{\prime }(t)\to 0$ as t→∞. For instance, it will not affect fall-offs according to power-laws. This follows from ${\left(D_{\infty }t+\beta (t)\right)}^{\alpha }={\left(D_{\infty }t\right)}^{\alpha }{\left(1+\beta (t)/D_{\infty }t\right)}^{\alpha }$ and the fact that $\frac{\beta (t)}{D_{\infty }t}=o(1)$ as t→∞.

## III. A NEW FAMILY OF DISORDERS

Here, we first point out the properties of hyperuniform and the short-range disorders.^15^ We let $X∼S(a,\sigma^{2})$ indicate that *X* is a stochastic variable with mean *a* and variance $\sigma^{2}$, without explicit reference to the distribution *X* may belong to. Hence, if *X* and *Y* are two (possibly different) independent stochastic variables with $X∼S(a,\sigma^{2})$ and $Y∼S(b,\gamma^{2})$, then $X\pm Y∼S(a\pm b,\sigma^{2}+\gamma^{2})$.

For hyperuniform disorder, the membrane locations $x_{m}$ are samples of a variable $X_{m}∼S(mL,\sigma^{2}/2)$, where *L* is the mean separation. Note that $X_{n}$ and $X_{m}$ are independent if n≠m. We also note that (for k>0)

$$X_{m+k}-X_{m}∼S(kL,\sigma^{2}).$$ (23)

On the other hand, the membrane separations $L_{m}=X_{m}-X_{m-1}$ are not independent. Rather, $L_{n}$ and $L_{m}$ are dependent if m=n±1.

For short-range disorder, the increments $L_{m}$ are independent $L_{m}∼$ $S(L,\sigma^{2})$ for some σ, which means that the positions $X_{m}$ will be dependent. Hence, for k>0,

$$X_{m+k}-X_{m}=\sum_{n=m+1}^{k+m}\left(X_{n}-X_{n-1}\right)∼S(kL,k\sigma^{2}).$$ (24)

It should be noted that both cases are of the form

$$X_{m+k}-X_{m}∼S(kL,k^{\alpha }\sigma^{2}),$$ (25)

where α=1 for the short-range disorder while α=0 for hyperuniform disorder. It is possible to have dependencies as in (25) for other values of α. This requires (as for α=0) that the increments $L_{k}$ are correlated. In fact, it is possible to design disorder arrangements for α≤2 (with α=2 as a degenerate case, see Appendix C). This is achieved by letting the interval $L_{k}=X_{k}-X_{k-1}$ be dependent stochastic variables with prescribed covariances; a description is found in Appendix C.

## IV. RESULTS AND DISCUSSION

**FIG. 3.**
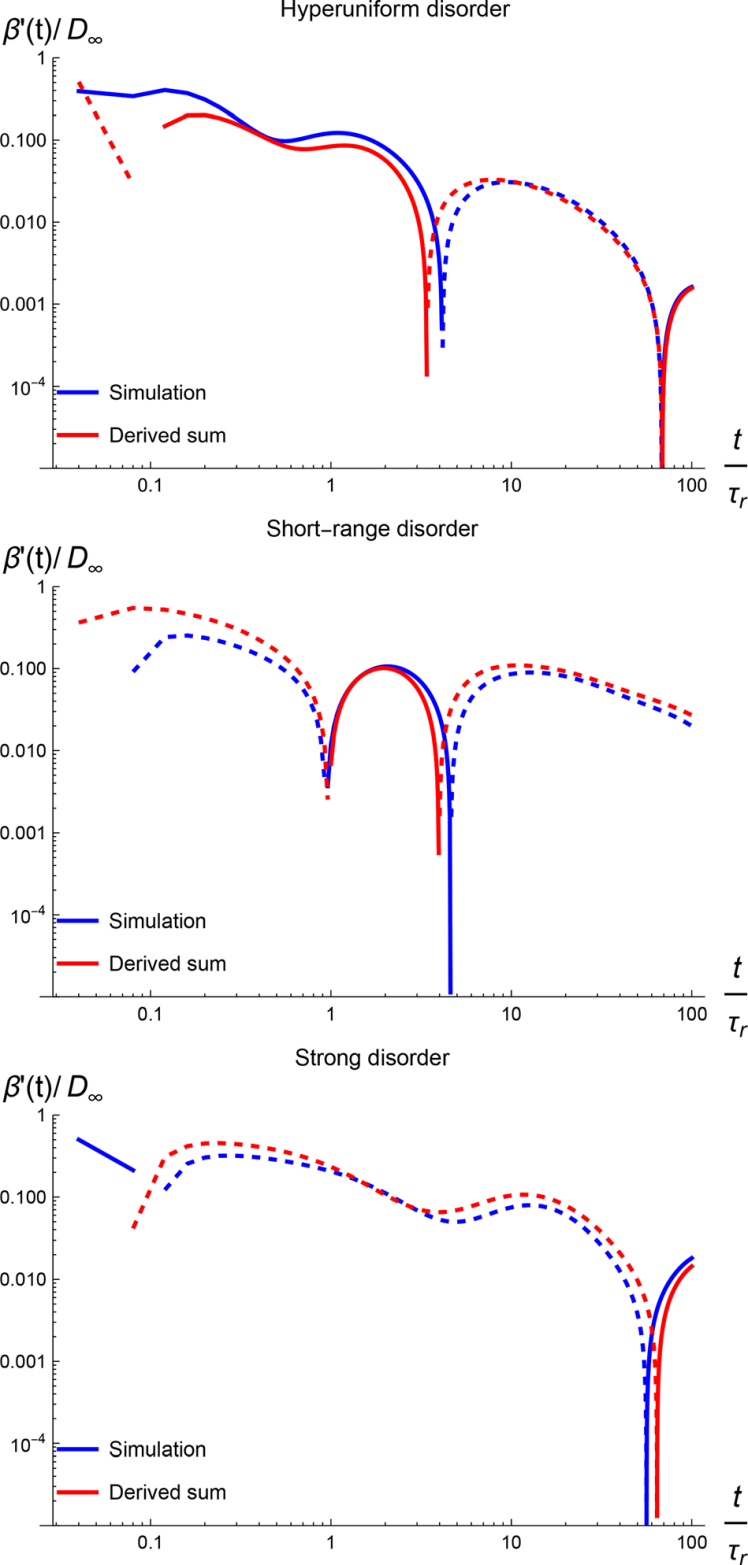
The convergence of the instantaneous diffusivity to its long-time value obtained through two independent methods. The dotted portions of the curves indicate negative values before taking the absolute values. The horizontal axis is made dimensionless by dividing the time by $\tau_{r}=L/2P$.

To assess our analytical derivation, we compared our results against those obtained through a finite-difference framework described in Appendix B. To this end, first, we considered realizations of system configurations exhibiting hyperuniform disorder, short-range disorder, and strong disorder.^15^ Note that for disordered media, the positions $x_{k}$ in Eq. (22) become stochastic variables. $D_{0},P$, and *L* are chosen so that dimensionless parameter $D_{0}/PL$ is set to 1. For the hyperuniform disorder, the stochastic membrane dislocation from the underlying regular grid is chosen from a uniform distribution with mean 0 and variance $\sigma^{2}=0.08L^{2}$. For the short-range disorder, the same applies except that now the interval length is from a uniform distribution with mean *L* and variance $\sigma^{2}=0.5L^{2}$. For the strong disorder, the disorder parameter^15^ was taken to be μ=1.75. The spatial range of the simulations was 100*L*, i.e., we considered scenarios with ∼100 membranes. For the finite-difference tool (see Appendix B), the above applies with the following addition. We chose dx=L/125 and $dt={\left(dx\right)}^{2}/2D_{0}$. For fixed *t*, with achieved simulated profile u(x,t) (known at $x=x_{k}=\text{kdx}$ for appropriate integers *k*), we numerically evaluate the mean-squared displacement to get 2Δ(t), where $\Delta (t)=D_{\infty }t+\beta (t)$. Finally, a numerical differentiation of Δ(t) together with the knowledge of $D_{\infty }$ gives $\beta^{\prime }(t)$. Figure 3 illustrates the results for each of these disorders. As expected, the two methods provide consistent results at long times. The slight discrepancies could be due to the approximation of Δ(t) as described in our remark following Eq. (22).

**FIG. 4.**
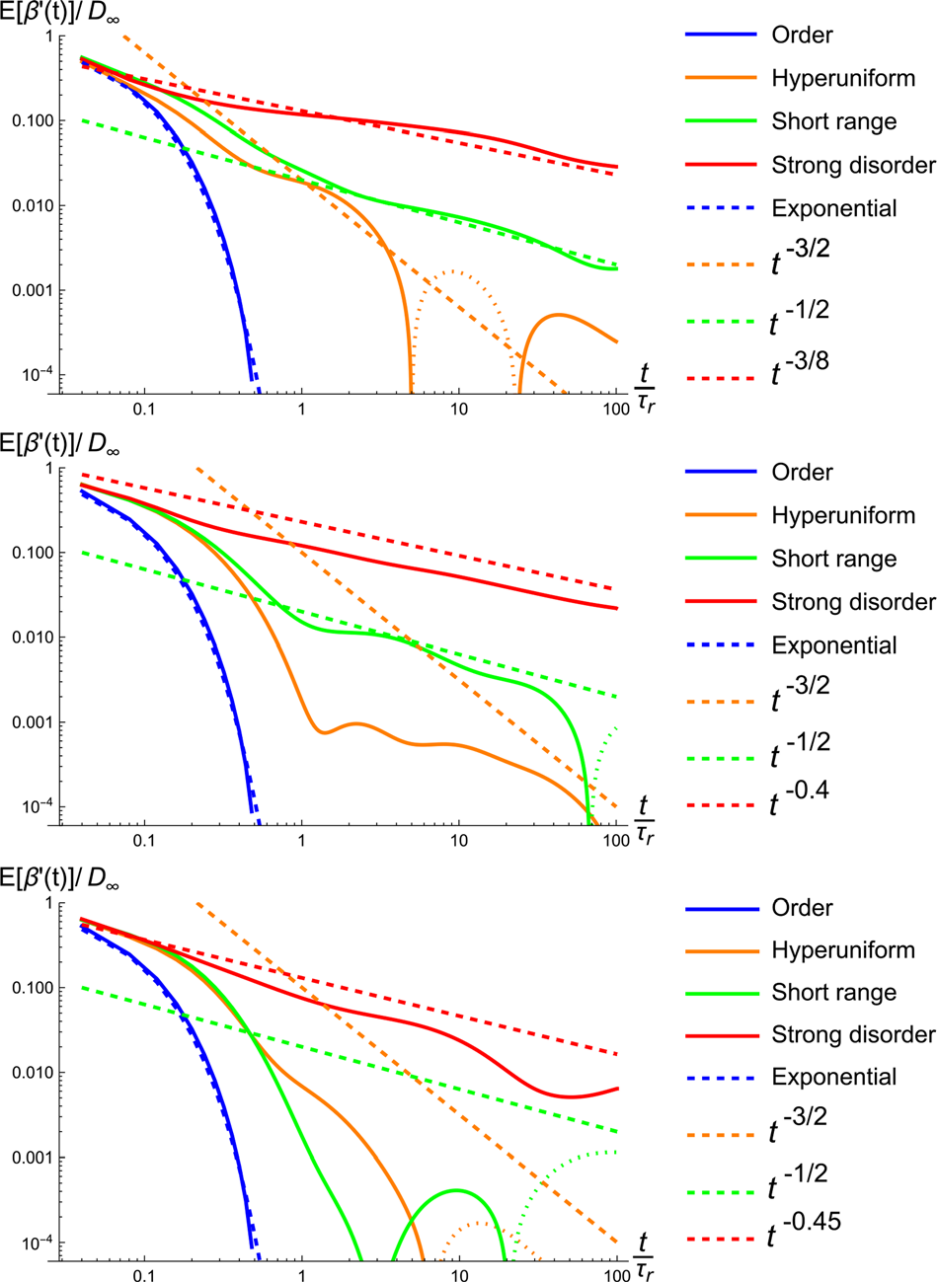
The approach of the instantaneous diffusivity to its long-time value for 200 realizations of each of the disorder classes considered. For all plots, $D_{0}/PL=1$. The disorder parameters were the following. Top: those from Fig. 3. Middle: $\sigma^{2}/L^{2}=0.02$ (hyperuniform), $\sigma^{2}/L^{2}=0.124$ (short range), μ=1.8 (strong disorder). Bottom: $\sigma^{2}/L^{2}=0.005$ (hyperuniform), $\sigma^{2}/L^{2}=0.031$ (short range), μ=1.9 (strong disorder). The dotted portions of the curves indicate negative values before taking the absolute values.

**FIG. 5.**
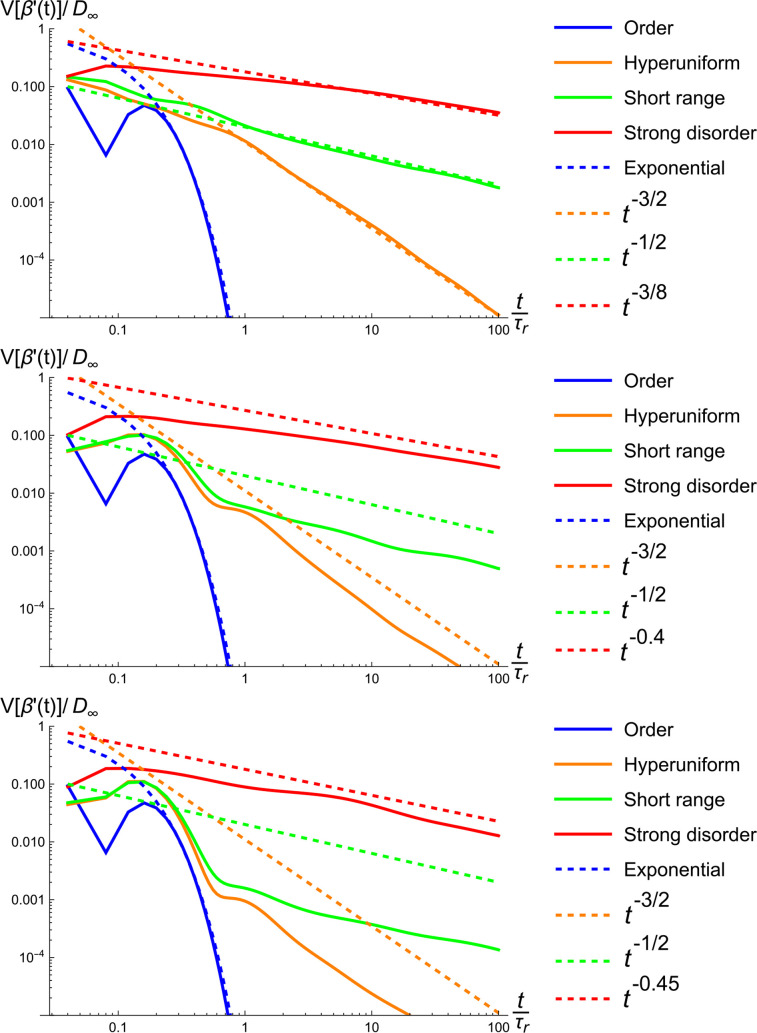
The variance of the deviation of $D_{\text{inst}}$ from $D_{\infty }$. The number of realizations and parameter setting are identical to those of Fig. 4. Interestingly, the fall-offs we observe for the variance are consistent with the power-laws reported in the literature^15^ for the expected values.

We also considered scenarios involving 200 different realizations for each of the disorder classes. These simulations are compared with the derived asymptotic formulas, and also with our analytical results. We find excellent agreement between our model and our finite-difference simulations; the latter results are shown in Figs. 4 and 5. In Fig. 4, we studied the deviation of $D_{\text{inst}}$ from $D_{\infty }$. The approach of $D_{\text{inst}}$ to $D_{\infty }$ appears to deviate somewhat from the power-law relations in the long-time regime suggested in the recent literature,^15^ most notably in the case of hyperuniform disorder. We also studied the variance of the quantity $(D_{\text{inst}}-D_{\infty })/D_{\infty }$, see Fig. 5. Interestingly, our results for the variance seem to exhibit the said power-laws. These findings are obtained by simulations of all disorder classes, and also the numerical evaluation of the sum above for the case of hyperuniform disorder.

**FIG. 6.**
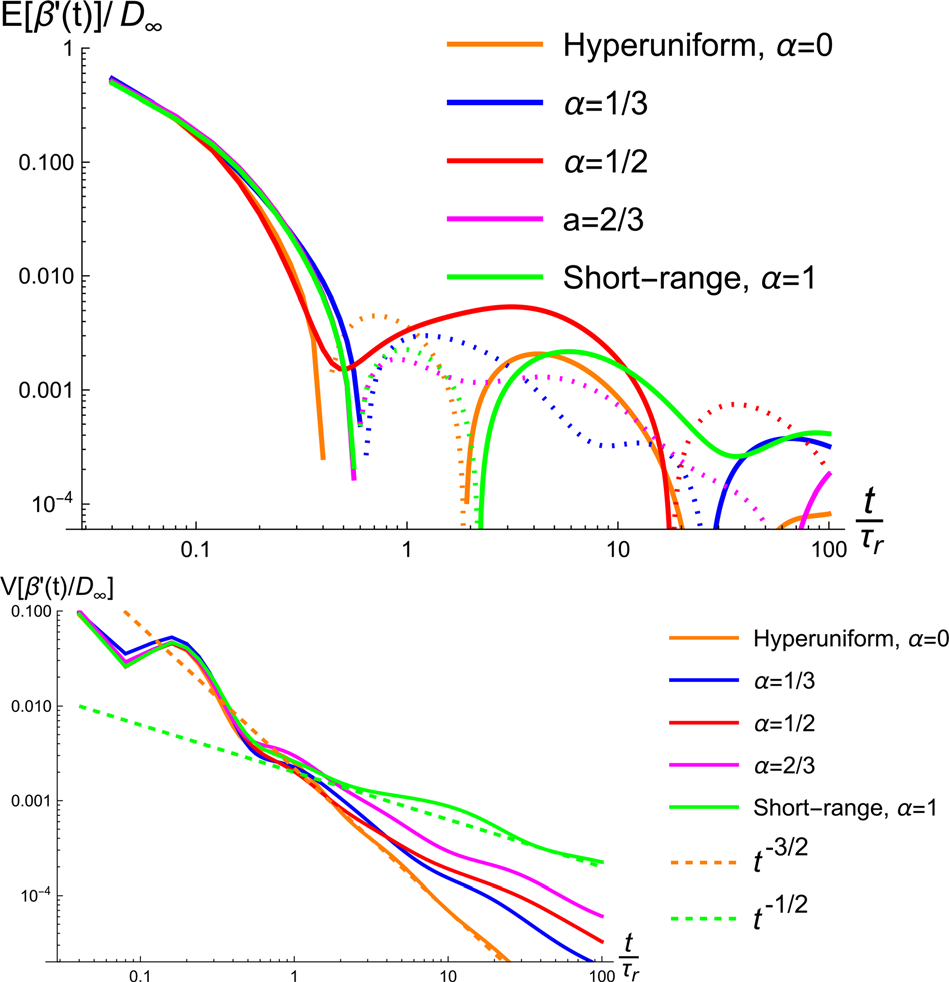
Top: The mean of 200 realizations each for five different disorder classes. Bottom: the variances of the same set of realizations. The disorder classes are from Sec. III with α=0,1/3,1/2,2/3,1.

In Sec. III, we introduced a new one parameter family of disorder classes, which goes from hyperuniform disorder ( α=0) to short-range disorder ( α=1). In Fig. 6, we show simulations for the expected value and variance of the quantity $(D_{\text{inst}}-D_{\infty })/D_{\infty }$ for this new disorder class with the parameter α taking the values 0,1/3,1/2,2/3,1.

The resulting formula (22) and the presented simulation tool can be generalized in different ways. Perhaps the most natural 1D extension of (22) is to make the membranes located at the positions $x_{k}$ having different permeabilities $P_{k}$. This could enter in Eq. (6), where *P* (put inside the summation) would be replaced with $P_{k}$. In a disorder scenario, where the membrane positions are stochastic variables, one may then make appropriate models for the statistics of the permeabilities. Also, varying diffusivity between plates may be considered.

We envision our approach being extended to higher dimensions, which would allow for accounting for the geometry at hand. Doing so would enable us to view diffusion anisotropy in a new light, especially in biological systems that have a myriad of membrane barriers, as potentially arising from different disorder classes along different directions or at least the same disorder classes but with different distributions of microstructural parameters (e.g., *L*s) or transport properties (e.g., *D*s and *P*s). It also provides a means for understanding the basis of the observed diffusion time dependence of different diffusion anisotropy measures.^25^

There are other ways in which this modeling framework can be meaningfully extended. One can consider domains having different (or alternating) diffusivities, such as the case in biological tissue with distinct intra- and extra-cellular compartments, each having different apparent diffusion coefficients (ADCs). This would make this modeling framework closer to describing MR methods used in biomedical applications, such as diffusion exchange spectroscopy (DEXSY), which describe translocation of water molecules from one diffusion domain to another.^26^ The effects of fluctuations in the particles' drift velocities^27^ or their changing diffusivities could be incorporated^28^ to account for dynamic spatiotemporal heterogeneities that could be present in living tissues.

In addition, the results of this model could be fed into simulations describing the phase history of spins, such as water protons, as a way to predict the nuclear magnetic resonance (NMR) signal produced with different assumed material morphologies and transport parameters. Relaxation could be added to describe the possible signal decay resulting from migration from one compartment to another. Surface relaxation could be included as well.

Finally, our results comport well with some previous findings and vary somewhat from others. Our simulation tool recapitulates the one-membrane result provided by Powles *et al.*,^23^ while our theoretical finding is consistent with the infinite-time diffusivity of Crick.^22^ However, the approach to this asymptotic limit at diffusion times longer than that simulated by Novikov *et al.* exhibits a deviation from the power-laws reported earlier.^15^ Interestingly, we do observe those power-laws for the variance of the realizations for each disorder class. This may be due to a difference in simplifying assumptions that the two models make.

## V. CONCLUSION

With our approach, we present a flexible modeling framework that is easy to understand and implement, which predicts the asymptotic instantaneous diffusivity for a one-dimensional structure with membrane locations prescribed by various statistical models. We expect our methods will improve our understanding of the relationship between the time-dependent diffusivity and material properties and porous media morphology. This work should ultimately help us infer material properties and microstructural features from measured diffusion magnetic resonance signals and images.

## Figures and Tables

**FIG. 7.**
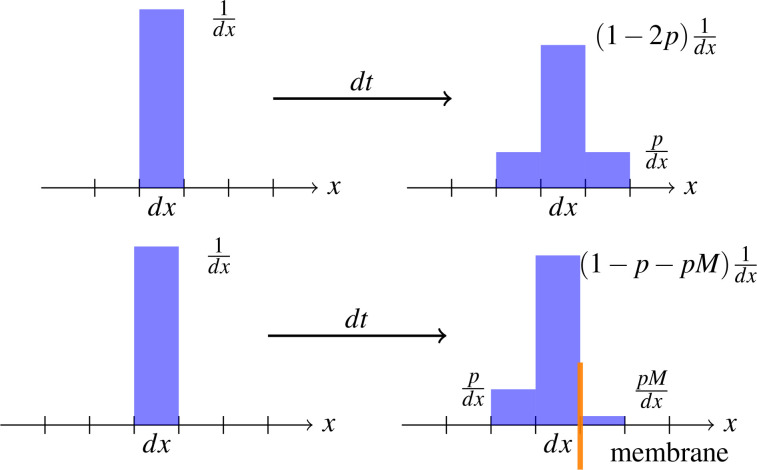
The discretization employed in the simulation framework provides an alternative means for computing the particle distribution without membranes (top) and with membranes (bottom). At each (small) time step, the probability of the particle leaks into neighboring bins. If a membrane is to be traversed, this probability is reduced. This evolution can be expressed as a matrix–vector multiplication, with the matrix being tri-diagonal. The distribution at any point in time is computed via repeated multiplications. This Markov modeling framework provides a powerful means to probe diffusion in one-dimensional disordered media.

**FIG. 8.**
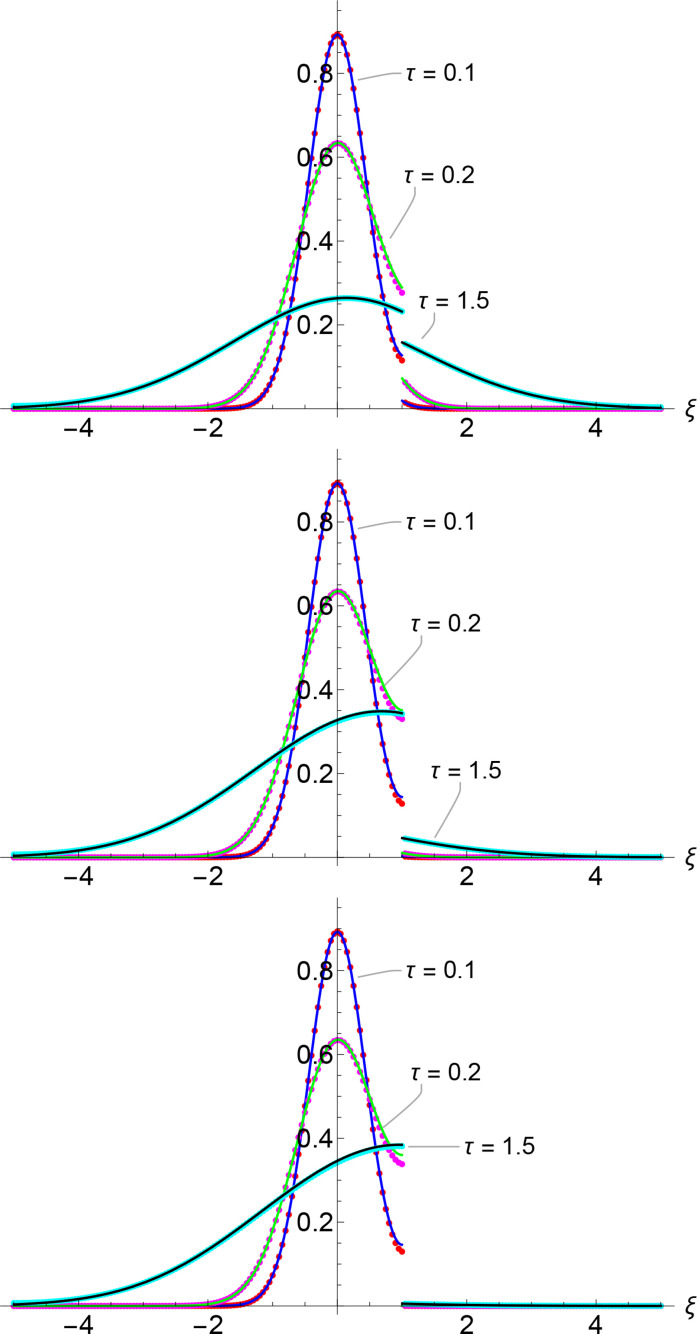
The results of the simulation tool (dots) compared with the analytical result provided in Eq. (B3) (solid lines) for τ=0.1, τ=0.2, and τ=1.5. The *R* values were taken to be R=0.01 (top), R=0.1 (middle), and R=1 (bottom).

## Data Availability

Data sharing is not applicable to this article as no new data were created or analyzed in this study.

## APPENDIX A: PROOF OF Eq. (17)

Here, we present a condensed/sketched proof of Eq. (17). By inserting a membrane at x = 0 and chose enumeration so that this point is $x_{0}$, by putting $T^{2}=\Delta (t)/(4L^{2})$ with T>0, and by replacing $x_{k}/L$ with $y_{k}$, the symmetry (in *x*) of *u* shows that we have to prove that, with $a_{k}=y_{k}^{2}/T^{2}e^{-y_{k}^{2}/T^{2}}$,

$$\frac{1}{T}\sum_{k=0}^{\infty }a_{k}=\frac{1}{T}\sum_{k=0}^{\infty }\frac{y_{k}^{2}}{T^{2}}e^{-\frac{y_{k}^{2}}{T^{2}}}\to \frac{\sqrt{\pi }}{4}\;\text{as}\;T\to \infty .$$ (A1)

The requirement that *L* is the mean separation, i.e., $L=\underset{N\to \infty }{\text{lim}}\frac{1}{N}\sum_{k=1}^{N}\left(x_{k}-x_{k-1}\right)$ translates into $\underset{k\to \infty }{\text{lim}}y_{k}/k=1$. We note that the function $f(x)=x^{2}e^{-x^{2}}$ is increasing on [0, 1] and decreasing on [1,∞] (with maximum value 1/e). Let ε>0. Then, there exists a *K* such that $1-\varepsilon <y_{k}/k<1+\varepsilon$, i.e., $(1-\varepsilon )k<y_{k}<(1+\varepsilon )k$ if k≥K. We now split the sum in (A1) into four parts

$$\frac{1}{T}\sum_{k=0}^{\infty }a_{k}=\frac{1}{T}\sum_{k=0}^{K-1}a_{k}+\frac{1}{T}\sum_{k=K}^{J_{1}-1}a_{k}+\frac{1}{T}\sum_{k=J_{1}}^{J_{2}}a_{k}+\frac{1}{T}\sum_{k=J_{2}+1}^{\infty }a_{k},$$ (A2)

where $J_{1}=\left\lfloor \frac{T}{1+\varepsilon }\right\rfloor ,\;J_{2}=\left\lfloor \frac{T}{1-\varepsilon }\right\rfloor$. It is clear that the first sum goes to 0 as T→∞. The third sum is bounded by (positive terms) $\frac{1}{T}(J_{2}-J_{1}+1)/e$, which is roughly $2\varepsilon /(1-\varepsilon^{2})$ for large *T*. In the second sum, it holds that

$$\frac{{\left(1-\varepsilon \right)}^{2}k^{2}}{T^{2}}e^{-\frac{{\left(1-\varepsilon \right)}^{2}k^{2}}{T^{2}}}<\frac{y_{k}^{2}}{T^{2}}e^{-\frac{y_{k}^{2}}{T^{2}}}<\frac{{\left(1+\varepsilon \right)}^{2}k^{2}}{T^{2}}e^{-\frac{{\left(1+\varepsilon \right)}^{2}k^{2}}{T^{2}}},$$ (A3)

while in the fourth sum, the inequalities are reversed. Viewed as a Riemann sum,

$$\frac{1}{T}\sum_{k=K}^{J_{1}-1}\frac{{\left(1-\varepsilon \right)}^{2}k^{2}}{T^{2}}e^{-\frac{{\left(1-\varepsilon \right)}^{2}k^{2}}{T^{2}}}\to \int_{0}^{\frac{1}{1+\varepsilon }}{\left(1-\varepsilon \right)}^{2}y^{2}e^{-{\left(1-\varepsilon \right)}^{2}y^{2}}dy,$$ (A4)

as T→∞ and similarly

$$\frac{1}{T}\sum_{k=K}^{J_{1}-1}\frac{{\left(1+\varepsilon \right)}^{2}k^{2}}{T^{2}}e^{-\frac{{\left(1+\varepsilon \right)}^{2}k^{2}}{T^{2}}}\to \int_{0}^{\frac{1}{1+\varepsilon }}{\left(1+\varepsilon \right)}^{2}y^{2}e^{-{\left(1+\varepsilon \right)}^{2}y^{2}}dy.$$ (A5)

If ε is sufficiently small, both these integrals differ from $\int_{0}^{1}y^{2}e^{-y^{2}}dy$ with less than Cε for some *C* (which does not depend on ε). By similar arguments for the fourth sum, the conclusion is that there exists a constant *K* such that

$$\left|\frac{1}{T}\sum_{k=0}^{\infty }\frac{y_{k}^{2}}{T^{2}}e^{-\frac{y_{k}^{2}}{T^{2}}}-\int_{0}^{\infty }y^{2}e^{-y^{2}}dy\right|<K\varepsilon ,$$ (A6)

if *T* is large enough. Since $\int_{0}^{\infty }y^{2}e^{-y^{2}}dy=\sqrt{\pi }/4$, (A1) follows.

## APPENDIX B: THE SIMULATION TOOL

We employed an independent implementation of a finite-difference simulation framework,^29–32^ which is summarized in Fig. 7. The domain is divided into equal-sized bins, and the particle concentration profile, which is initially confined to one such bin, is expressed as a vector v. The time evolution for a small time-interval is expressed as a matrix product v→Av, where the matrix *A* captures the diffusion process during a time step *dt*, taking into consideration the possible exchange through the membrane with permeability *P*. Repeated multiplication by *A* allows for estimation of the particle distribution at any time.

Due to the nearest neighbor model, the matrix *A* becomes tri-diagonal, hence the matrix-vector multiplication is very fast. The diagonal elements of *A* are to be taken (1−p−pM)/dx, while the off-diagonals are taken to be p/dx and pM/dx, where *dx* is the size of the bins, *dt* is the time step, and

$$p=\frac{D_{0}dt}{{\left(dx\right)}^{2}},\;M=\frac{\text{dxP}}{D_{0}+\text{dxP}}.$$ (B1)

Note that in the absence of membranes, i.e., if P=∞, then M=1.

These values for *p* and *M* as well as our implementation of this finite-difference framework are supported by plots comparing the simulation tool with the known explicit solution^23^ for the case when there is just one membrane at x=L>0

$$\begin{array}{c} u(x,t)=\{\begin{array}{cc} \frac{e^{-\frac{x^{2}}{4Dt}}}{\sqrt{4\pi Dt}}+\frac{e^{-\frac{{\left(x-2L\right)}^{2}}{4Dt}}}{\sqrt{4\pi Dt}}-\frac{P}{D}e^{\frac{2P(2L+2Pt-x)}{D}}\text{erfc}\left(\frac{2L+4Pt-x}{2\sqrt{Dt}}\right) & x<L\\ \frac{P}{D}e^{\frac{2P(2Pt+x)}{D}}\text{erfc}\left(\frac{4Pt+x}{2\sqrt{Dt}}\right) & x>L \end{array}, \end{array}$$ (B2)

where erfc is the complementary error function. To validate the choice of *M* using (B2), it is convenient to introduce dimensionless quantities ξ=x/L, $\tau =Dt/L^{2}$, and R=LP/D, after which *u* in (B2) becomes

$$\begin{array}{c} Lu(\xi ,\tau )=\{\begin{array}{cc} \frac{e^{-\frac{\xi^{2}}{4\tau }}}{\sqrt{4\pi \tau }}+\frac{e^{-\frac{{\left(\xi -2\right)}^{2}}{4\tau }}}{\sqrt{4\pi \tau }}-Re^{2R(2+2R\tau -\xi )}\text{erfc}\left(\frac{2+4R\tau -\xi }{2\sqrt{\tau }}\right) & \xi <1\\ Re^{2R(2R\tau +\xi )}\text{erfc}\left(\frac{4R\tau +\xi }{2\sqrt{\tau }}\right) & \xi >1. \end{array} \end{array}$$ (B3)

In Fig. 8, we compare the outcome of the simulation tool for R=0.01, R=0.1, and R=1 with the function (B3). For each case, the density function is evaluated for τ=0.1, τ=0.2, and τ=1.5.

## APPENDIX C: ON THE NEW FAMILY OF DISORDERS

In this appendix, we justify the claims from Sec. III. We first note that

$$X_{m+k}-X_{m}=\sum_{n=m+1}^{k+m}\left(X_{n}-X_{n-1}\right)=\sum_{n=m+1}^{k+m}L_{n},$$ (C1)

so that (25) can be expressed in terms of the interval lengths $L_{k}$. We remind that $X∼S(L,\sigma^{2})$ indicates that *X* is a stochastic variable with mean *L* and variance $\sigma^{2}$.

**Lemma 1**. *Suppose that the stochastic variables* $L_{m}∼S(L,\sigma^{2}),\;m\in Z$*. Suppose further that for all m and for some* 0≤α≤2, $\text{Cov}(L_{m},L_{m+1})=(2^{\alpha -1}-1)\sigma^{2}$ *and that for* k≥2, $\text{Cov}(L_{m},L_{m+k})=\frac{1}{2}({\left(k-1\right)}^{\alpha }-2k^{\alpha }+{\left(k+1\right)}^{\alpha })\sigma^{2}$*. Then*

$$\sum_{n=m+1}^{k+m}L_{n}∼S(kL,k^{\alpha }\sigma^{2}).$$ (C2)

*Proof*. Due to symmetry, $\text{Var}(\sum_{n=m+1}^{k+m}L_{n})=\sum_{n=m+1}^{k+m}\text{Var}(L_{n})+2\sum_{j=1}^{k-1}\sum_{n=m+1}^{k+m-j}\text{Cov}(L_{n},L_{n+j}).$ This can be written

$$\begin{array}{c} k\text{Var}(L_{k})+2\sum_{j=1}^{k-1}\left(k-j\right)\text{Cov}(L_{k},L_{k+j})\\ =k\sigma^{2}+(k-1)(2^{\alpha }-2)\sigma^{2}\\ \;+\sum_{j=2}^{k-1}\left(k-j\right)({\left(j-1\right)}^{\alpha }-2j^{\alpha }+{\left(j+1\right)}^{\alpha })\sigma^{2}\\ =k(2^{\alpha }-1)\sigma^{2}+(2-2^{\alpha })\sigma^{2}\\ \;+\sum_{j=2}^{k-1}\left(k-j\right)({\left(j-1\right)}^{\alpha }-2j^{\alpha }+{\left(j+1\right)}^{\alpha })\sigma^{2}. \end{array}$$ (C3)

By splitting the sum and changing the summation index in two of the sums, we arrive at

$$\begin{array}{c} k(2^{\alpha }-1)\sigma^{2}+(2-2^{\alpha })\sigma^{2}+\left[\sum_{j=1}^{k-2}\left(k-j-1\right)j^{\alpha }-2\sum_{j=2}^{k-1}\left(k-j\right)j^{\alpha }+\sum_{j=3}^{k}\left(k-j+1\right)j^{\alpha }\right]\sigma^{2}=(k(2^{\alpha }-1)+(2-2^{\alpha })+(k-2)+(k-3)2^{\alpha }-2(k-2)2^{\alpha }-2{\left(k-1\right)}^{\alpha }+2{\left(k-1\right)}^{\alpha }+k^{\alpha })\sigma^{2}=k^{\alpha }\sigma^{2}. \end{array}$$ (C4)

□

Put, for n≥0 and any *m*, $c_{n}=\text{Cov}(L_{m},L_{m+n})$, i.e., $c_{0}=\sigma^{2}$, $c_{1}=(2^{\alpha -1}-1)\sigma^{2}$, and $c_{k}=\frac{1}{2}({\left(k-1\right)}^{\alpha }-2k^{\alpha }+{\left(k+1\right)}^{\alpha })\sigma^{2}$ for k≥2. We then note that α=1 implies $c_{k}=0$ for k≥1. This means that the increments are independent, which is the defining feature of the short-range disorder. On the other hand, α=0 gives $c_{1}=-1/2\sigma^{2}$, $c_{k}=0$ for k≥2, which is in agreement with the hyperuniform disorder, where only neighboring intervals are correlated (since they share a membrane point).

For the infinite sequence of membrane separations $L_{k}$, the shift invariance of the covariances makes the auto correlation function important. However, in practice to simulate diffusion within a particular realization, we study a large but finite number of intervals, in which case we can also inspect the covariance matrix *C*. For α=1, we have

$$C=\sigma^{2}I,$$ (C5)

where *I* is the identity matrix of the appropriate size. For α=0, we have

$$\begin{array}{c} C=\sigma^{2}\left(\begin{array}{cccccc} 1 & -1/2 & 0 & \cdots & 0 & 0\\ -1/2 & 1 & -1/2 & 0 & \cdots & 0\\ 0 & -1/2 & 1 & -1/2 & \cdots & 0\\ 0 & & & \ddots & & \vdots \\ \vdots & & & & & -1/2\\ 0 & & & \cdots & -1/2 & 1 \end{array}\right). \end{array}$$ (C6)

It is also interesting to examine the degenerate case α=2. Then, all $c_{k}=1$ for k=0,1,2…, which gives a covariance matrix with all entries $\sigma^{2}$. This matrix has rank 1, and all intervals are completely correlated so that $\text{Var}(\sum_{n=m+1}^{k+m}L_{n})=k^{2}\sigma^{2}.$

Note also that values α>2 are ruled out since *C* will not be positive semi-definite. The more interesting cases are when α<1 (or possibly α<2), together with the question of realization of such membrane disorders.

There is no loss of generality to consider $L_{m}∼S(0,1)$ since we can always scale with $\sigma^{2}$ and also add the mean length *L* afterward. Hence, by putting a finite number of variables $L_{n}$ in a vector L of length *K*, the covariance condition says

$$E[LL^{t}]=C=\left(\begin{array}{cccccc} 1 & c_{1} & c_{2} & \cdots & c_{K-2} & c_{K-1}\\ c_{1} & 1 & c_{1} & c_{2} & \cdots & c_{K-2}\\ c_{2} & c_{1} & 1 & c_{1} & \cdots & c_{K-3}\\ c_{3} & & & \ddots & & \vdots \\ \vdots & & & & & \vdots \\ c_{K-1} & & & \cdots & & 1 \end{array}\right).$$ (C7)

*C* can be diagonalized so that $C=UDU^{t}$, where *D* has the (non-negative) eigenvalues on the diagonal and *U* has the (normalized) eigenvectors as columns. In particular, $UU^{t}=I$. Then

$$E[U^{t}LL^{t}U]=U^{t}CU=U^{t}UDU^{t}U=D.$$ (C8)

With $Z=U^{t}L$, we have

$$E[ZZ^{t}]=D.$$ (C9)

However, this is easily produced. If the diagonal matrix *D* has eigenvalues $\lambda_{1},\lambda_{2},\ldots ,\lambda_{K}$ as elements, Z should be a vector of independent variables where $Z_{k}∼S(0,\sigma^{2}=\lambda_{k})$. The increments $L_{k}$ are then found in the vector L=UZ. After scaling with $\sigma^{2}$ and addition of the mean length *L*, one can produce membrane realization.

## References

[1] J. W. Haus and K. W. Kehr, “Diffusion in regular and disordered lattices,” Phys. Rep. 150, 263–406 (1987). 10.1016/0370-1573(87)90005-6

[2] D. Ben-Avraham and S. Havlin, *Diffusion and Reactions in Fractals and Disordered Systems* (Cambridge University Press, Cambridge, UK, 2000).

[3] J. E. Tanner, “Transient diffusion in a system partitioned by permeable barriers. Application to NMR measurements with a pulsed field gradient,” J. Chem. Phys. 69, 1748–1754 (1978). 10.1063/1.436751

[4] P. P. Mitra, P. N. Sen, and L. M. Schwartz, “Short-time behavior of the diffusion coefficient as a geometrical probe of porous media,” Phys. Rev. B 47, 8565 (1993). 10.1103/PhysRevB.47.8565 10004895

[5] L. L. Latour, P. P. Mitra, R. L. Kleinberg, and C. H. Sotak, “Time-dependent diffusion coefficient of fluids in porous media as a probe of surface-to-volume ratio,” J. Magn. Reson. Ser. A 101, 342–346 (1993). 10.1006/jmra.1993.1056

[6] T. M. de Swiet and P. N. Sen, “Time dependent diffusion coefficient in a disordered medium,” J. Chem. Phys. 104, 206–209 (1996). 10.1063/1.470890

[7] P. N. Sen, “Time-dependent diffusion coefficient as a probe of geometry,” Concepts Magn. Reson. Part A 23A, 1–21 (2004). 10.1002/cmr.a.20017

[8] P. N. Sen, L. M. Schwartz, P. P. Mitra, and B. I. Halperin, “Surface relaxation and the long-time diffusion coefficient in porous media: Periodic geometries,” Phys. Rev. B 49, 215 (1994). 10.1103/PhysRevB.49.215 10009277

[9] L. L. Latour, K. Svoboda, P. P. Mitra, and C. H. Sotak, “Time-dependent diffusion of water in a biological model system,” Proc. Natl. Acad. Sci. 91, 1229–1233 (1994). 10.1073/pnas.91.4.1229 8108392 PMC43130

[10] P. N. Sen, “Time-dependent diffusion coefficient as a probe of the permeability of the pore wall,” J. Chem. Phys. 119, 9871–9876 (2003). 10.1063/1.1611477

[11] O. K. Dudko, A. M. Berezhkovskii, and G. H. Weiss, “Time-dependent diffusion coefficients in periodic porous materials,” J. Phys. Chem. B 109, 21296–21299 (2005). 10.1021/jp051172r 16853761

[12] A. L. Sukstanskii, D. A. Yablonskiy, and J. J. H. Ackerman, “Effects of permeable boundaries on the diffusion-attenuated MR signal: Insights from a one-dimensional model,” J. Magn. Reson. 170, 56–66 (2004). 10.1016/j.jmr.2004.05.020 15324758

[13] A. Klemm, R. Metzler, and R. Kimmich, “Diffusion on random-site percolation clusters: Theory and NMR microscopy experiments with model objects,” Phys. Rev. E 65, 021112 (2002). 10.1103/PhysRevE.65.021112 11863508

[14] E. Özarslan, P. J. Basser, T. M. Shepherd, P. E. Thelwall, B. C. Vemuri, and S. J. Blackband, “Observation of anomalous diffusion in excised tissue by characterizing the diffusion-time dependence of the MR signal,” J. Magn. Reson. 183, 315–323 (2006). 10.1016/j.jmr.2006.08.009 16962801

[15] D. S. Novikov, J. H. Jensen, J. A. Helpern, and E. Fieremans, “Revealing mesoscopic structural universality with diffusion,” Proc. Natl. Acad. Sci. U. S. A. 111, 5088–5093 (2014). 10.1073/pnas.1316944111 24706873 PMC3986157

[16] S. Alexander, J. Bernasconi, W. Schneider, and R. Orbach, “Excitation dynamics in random one-dimensional systems,” Rev. Mod. Phys. 53, 175 (1981). 10.1103/RevModPhys.53.175

[17] M. J. Stephen and R. Kariotis, “Diffusion in a one-dimensional disordered system,” Phys. Rev. B 26, 2917 (1982). 10.1103/PhysRevB.26.2917 9992012

[18] M. Mobilia and P.-A. Bares, “Solution of a class of one-dimensional reaction-diffusion models in disordered media,” Phys. Rev. B 64, 064203 (2001). 10.1103/PhysRevB.64.064203

[19] P. L. Doussal and C. Monthus, “Reaction diffusion models in one dimension with disorder,” Phys. Rev. E 60, 1212 (1999). 10.1103/PhysRevE.60.1212 11969881

[20] Y. A. Berlin and A. L. Burin, “Diffusion in one-dimensional disordered systems,” Chem. Phys. Lett. 257, 665–673 (1996). 10.1016/0009-2614(96)00597-0

[21] R. Metzler and J. Klafter, “The random walk's guide to anomalous diffusion: A fractional dynamics approach,” Phys. Rep. 339, 1–77 (2000). 10.1016/S0370-1573(00)00070-3

[22] F. Crick, “Diffusion in embryogenesis,” Nature 225, 420–422 (1970). 10.1038/225420a0 5411117

[23] J. G. Powles, M. Mallett, G. Rickayzen, and W. Evans, “Exact analytic solutions for diffusion impeded by an infinite array of partially permeable barriers,” Proc. R. Soc. London, Ser. A 436, 391–403 (1992). 10.1098/rspa.1992.0025

[24] With well-defined mean separation.

[25] E. Özarslan, T. M. Shepherd, C. G. Koay, S. J. Blackband, and P. J. Basser, “Temporal scaling characteristics of diffusion as a new MRI contrast: Findings in rat hippocampus,” NeuroImage 60, 1380–1393 (2012). 10.1016/j.neuroimage.2012.01.105 22306798 PMC3303993

[26] P. T. Callaghan and I. Furó, “Diffusion-diffusion correlation and exchange as a signature for local order and dynamics,” J. Chem. Phys. 120, 4032–4038 (2004). 10.1063/1.1642604 15268569

[27] A. M. Berezhkovskii and A. Szabo, “Effective diffusivity for transport with fluctuating drift velocity,” J. Phys. Chem. B 125, 4489–4493 (2021). 10.1021/acs.jpcb.1c01856 33881851

[28] Y. Lanoiselée, N. Moutal, and D. S. Grebenkov, “Diffusion-limited reactions in dynamic heterogeneous media,” Nat. Commun. 9, 4398 (2018). 10.1038/s41467-018-06610-6 30353010 PMC6199324

[29] C. A. Fletcher, *Computational Techniques for Fluid Dynamics*, Fundamental and General Techniques Vol. I (Springer Verlag, New York, 1991).

[30] C.-L. Chin, F. W. Wehrli, S. N. Hwang, M. Takahashi, and D. B. Hackney, “Biexponential diffusion attenuation in the rat spinal cord: Computer simulations based on anatomic images of axonal architecture,” Magn. Reson. Med. 47, 455–460 (2002). 10.1002/mrm.10078 11870831

[31] V. Aho, K. Mattila, T. Kühn, P. Kekäläinen, O. Pulkkinen, R. B. Minussi, M. Vihinen-Ranta, and J. Timonen, “Diffusion through thin membranes: Modeling across scales,” Phys. Rev. E 93, 043309 (2016). 10.1103/PhysRevE.93.043309 27176430

[32] M. Herberthson, E. Özarslan, and P. J. Basser, “Time-dependent diffusion in one-dimensional disordered media decorated by permeable membranes,” in *Proceedings of the 33rd Annual Meeting of ISMRM* (ISMRM, 2024), p. 3461. 10.1063/5.0272370 PMC12207535 40599743

